# A possible mechanism of biological silicification in plants

**DOI:** 10.3389/fpls.2015.00853

**Published:** 2015-10-09

**Authors:** Christopher Exley

**Affiliations:** The Birchall Centre, Lennard-Jones Laboratories, Keele UniversityStoke-on-Trent, UK

**Keywords:** plant silica, silicic acid, biological silicification, bioinorganic chemistry

## Abstract

Plants are significant exponents of biological silicification. While not all plants are generally considered as biosilicifiers the extent to which all plants deposit biogenic silica is largely unknown. There are plants which are known as silica accumulators though even in these plants the extent and degree to which their tissues are silicified is neither appreciated nor understood. An elucidation of the mechanism of silicification in biota is complicated by a lack of known bio-organic chemistry of silicic acid, the starting point in this process. Herein I argue the case that biological silicification is an entirely passive process. It is passive from the point of view that its underlying mechanisms and processes do not require us to invoke any as yet undiscovered silicon biochemistry. It is also passive in that although silicification confers clear biological/ecological advantages under certain conditions, it is actually non-essential in all plants and potentially, at least, toxic in some.

## Silicon is Taken Up by Plants as Silicic Acid

The only form of silicon in soil waters which is available for entry or uptake into a plant is silicic acid, Si(OH)_4_ (Exley, 1998). Its molecular structure is a single atom of silicon surrounded in a tetrahedral configuration by four hydroxyl (or silanol) groups. The pka for Si(OH)_4_ is *ca.* 9.6 which means that it is neutral under almost all possible soil water milieus and that it does not lose its first proton from any of the hydroxyl groups at pH below 10. Therefore Si(OH)_4_ is a very weak acid and additionally where conditions do allow for its effective deprotonation (pH > 10) any purported monosilicate species are expected to be unstable and to immediately form disilicate anions (Exley and Sjöberg, 2014). The disilicate anion, Si_2_O_2_(OH)_6_^2-^_(aq)_, which is a more stable form of silicate in aqueous media, is a precursor to ‘cement chemistry’ and through interactions with essential metal cations including calcium and magnesium would prove to be extremely toxic to plant roots should it be present in soil solutions or at root surfaces, so it too can be ruled out as a biologically available form of soil water silicon.

When considering the mechanism of biological silicification in plants it is imperative to recognize that there are simply no known circumstances where the form of silicon entering the root, or moving throughout the plant, is expected to be ‘silicate,’ SiO(OH)_3_^-^_(aq)_, and so all mechanisms which suggest a role for this form of silicon in silicon uptake and movement in plants should be re-evaluated. There are no known ligands for monosilicate anions in any transporters or channels that have previously been implicated in the uptake and movement of silicon. If the movement of silicon across a membrane does not involve any binding of silicon or even other silicon-specific interactions then this movement of silicon must be entirely passive. The seminal works by Ma et al. (2006, 2007) and Yamaji et al. (2008) on the movement of silicon in plants have inadvertently confused this subject by their suggestions that they have identified silicon transporters. These important pieces of research have actually identified channels or pores through which silicon as Si(OH)_4_ can pass by an unidentified but almost certainly passive mechanism. It is important to emphasize that the subject of biochemistry differentiates quite clearly between channels and transporters and it is commonly and widely accepted in biochemical nomenclature that transporters mediate (or catalyze) the movement of a solute by physically binding to the solute in facilitating its movement across a membrane. The binding of Si(OH)_4_, or any other form of silicon, has neither been demonstrated nor is known to occur in its movement across biological membranes. There are no known silicon transporters, as opposed to channels, in plants and the question then arises as to whether there are silicon-specific channels involved in the transmembrane passage of silicon? For example, experiments in which purported silicon transporters were expressed in oocytes and different rates of silicon movement through these channels were demonstrated depending upon the ambient conditions does not necessarily change such channels into transporters (Ma et al., 2006, 2007). It is still Si(OH)_4_ which moves across the membrane and there are other factors, not specifically or necessarily related to silicon, which have influenced its passive movement into oocytes and similarly its uptake and movement into and throughout plants. We shall visit these special circumstances throughout this essay. It is important to recognize at this point that previous research interpreted gradients of total silicon across biological membranes as evidence for active transport of silicon (Ma et al., 2006, 2007; Yamaji et al., 2008). Herein these observations are not discounted but re-interpreted with an emphasis on how they might be explained by passive movement of Si(OH)_4_.

## Silicic Acid Follows Water into Plants

Occam’s razor tells us quite categorically that the biologically available form of silicon in soil waters is Si(OH)_4_. Since this is a small neutral molecule with no known biochemical interactions with organic ligands and only a very limited inorganic chemistry there is a strong likelihood that the entry of Si(OH)_4_ into plant roots follows water (Raven, 1993; Epstein, 1994). This suggests two immediate pathways for the entry of Si(OH)_4_, an apoplastic or extracellular route, in which Si(OH)_4_ passes between cells, and a symplastic or intercellular route which will involve cell to cell movement of Si(OH)_4_. While the former may be the major pathway for the entry of Si(OH)_4_ into xylem it is also likely that both this process and the further movement of Si(OH)_4_ throughout xylem and other conducting tissue will involve some degree of movement of Si(OH)_4_ across biological membranes. If as is suggested herein that Si(OH)_4_ follows water then it will also use water channels or aquaporins as gateways across membranes. Indeed the so-called silicon transporters identified by Ma et al. (2002) are aquaporins with no specific selectivity for Si(OH)_4_ (Mitani et al., 2008). Aquaporins are a diverse set of water channels; note they are not transporters, with as many as 30 or more different forms in many plants (Li et al., 2014). These different forms, through their structural arrangements in membranes, present a continuum of selectivity toward the transmembrane passage of dissolved (physical and chemical) solutes (Ludewig and Dynowski, 2009) and will not only influence the ease with which Si(OH)_4_ crosses membranes but potentially help to establish concentration gradients of Si(OH)_4_ between connecting compartments. The flow of water into and throughout a plant is primarily driven by hydrostatic pressure and so the entry of Si(OH)_4_ into a plant and its movement toward the extremities of a plant will likewise be under the influence of water flow and not simply osmosis (Knipfer and Fricke, 2011). While aquaporins do not offer significant resistance to the movement of water into and throughout a plant they are likely to be more resistive in the case of the much larger Si(OH)_4_ molecule (Ludewig and Dynowski, 2009) with some channels allowing easier passage of this molecule than others. This selective permeability or resistance of aquaporins (and potentially other solvent/solute channels) toward the movement of Si(OH)_4_ presents a mechanism to describe its concentration (relative to soil water), and potentially its super-saturation (>2 mM), within specific compartments.

## Plants are Permeable to Silicic Acid

The concentration of Si(OH)_4_ in soil water will be significantly below its solubility of *ca*. 2 mM. As a small neutral solute, Si(OH)_4_ will be carried by water across the relatively porous root cell wall and into the root and plant (Raats, 2007). Water flow, driven by hydrostatic forces, will enable the further movement of Si(OH)_4_ throughout the plant potentially culminating in its elimination from the plant through guttation (Yamaji et al., 2008). It is significant that the knockdown of a gene for an aquaporin involved in the movement of silicon into shoots resulted in the enhanced elimination of SiOH)_4_ from rice by guttation (Yamaji et al., 2008). Apoplastic water flow will ensure a continuous supply of Si(OH)_4_ to the plant, primarily via xylem, while osmosis and symplastic water flow will deliver Si(OH)_4_ to all regions and tissues of the plant. The movement of water into and throughout a plant involves its passage across membranes and these barriers offer varying degrees of resistance to water flow (Knipfer et al., 2011). Resistance is lowered by membrane channels known as aquaporins which are permeable to water. They are also permeable to the passive movement of solutes of sufficiently small size including Si(OH)_4_. The permeability of aquaporins to different solutes depends upon the relative sizes of the solutes and the channel pores and, for non-apoplastic flow, additionally the maintenance of a concentration gradient of some sort across the membrane. Following such routes Si(OH)_4_ moves freely throughout the plant from root to shoot. Theoretically where water goes, Si(OH)_4_ has the possibility of going there too. The absence of biological silicification cannot be construed to infer the absence of Si(OH)_4_ only the absence of conditions which would allow for its auto condensation and precipitation as biogenic silica. The uptake and movement of Si(OH)_4_ throughout the plant does not require silicon transporters *per se* only membrane channels to allow for both the hydrostatic (hydraulic) and osmotic movement of Si(OH)_4_ between adjacent compartments. Wherever and by which pathways water moves throughout the plant the neutral solute Si(OH)_4_ has the potential to follow without the need to invoke unknown or novel inorganic or organic chemistry of Si(OH)_4_.

## A Prerequisite for Plant Silicification

Water is both the vehicle (solvent) and the delivery system for the distribution of Si(OH)_4_ throughout a plant. Guttation is conceivably the only mechanism for the exit of Si(OH)_4_ (as Si(OH)_4_) from a plant. Silicon enters a plant as an under-saturated solution of Si(OH)_4_ and yet it is found as amorphous hydrated silica within a plant. Biogenic silica cannot be formed spontaneously unless the concentration of Si(OH)_4_ in a plant exceeds its solubility limit of *ca.* 2 mM. I have previously defined biological silicification as:

Biosilicification: the movement of silicic acid from environments in which its concentration does not exceed its solubility (<2 mM) to intracellular or systemic compartments in which it is accumulated for subsequent deposition as amorphous hydrated silica (Exley, 2009b).

Thus the auto condensation of Si(OH)_4_ in a plant requires either a mechanism to concentrate Si(OH)_4_ above its solubility limit or a process whereby the barrier to its auto condensation can be lowered to enable the formation of biogenic silica at under-saturated concentrations of Si(OH)_4_. Arguably both of these mechanisms may be involved in silicification in plants.

Xylem should be considered as the major conduit for the movement of Si(OH)_4_ from root to shoot. Measurements of molybdate-reactive silicon have consistently demonstrated xylem exudates to be super-saturated with Si(OH)_4_ (Hartley and Jones, 1972; Casey et al., 2003; Liang et al., 2005, 2006; Mitani et al., 2005). Some caution is required in interpreting these values as the reduced molybdosilicic acid complex only obeys Beer’s law at concentrations up to *ca.* 0.2 mM and so measurements of up to 18 mM Si(OH)_4_ in xylem exudates would have required significant pre-dilution of samples (Coradin et al., 2004). However, complementary (Fernández Honaine et al., 2013) Si NMR studies have confirmed that, whatever the precise concentration, silicon in xylem exudates is Si(OH)_4_ and not biogenic silica or complexes of silicon (Casey et al., 2003; Mitani et al., 2005). Such super-saturated concentrations of Si(OH)_4_ in xylem sap should be considered as anomalous as they are expected to be thermodynamically unstable (Exley and Sjöberg, 2014). However, xylem fluid *in situ* in a living plant is not a static system, it is a non-equilibrium system, and its dynamic nature combined with the relatively slow kinetics of the auto condensation of Si(OH)_4_ under *in vivo* conditions does provide for an explanation of its super-saturation *in planta*. This concept is supported by the experimental observation that when xylem sap which contained a super-saturated concentration of Si(OH)_4_ was removed from a plant, thereby creating a static as opposed to non-equilibrium system, the *ex planta* concentration of Si(OH)_4_ rapidly fell toward its solubility maximum of *ca.* 2 mM (Mitani et al., 2005). This demonstrated that once the xylem fluid was outside of the plant it was not possible to maintain it as a super-saturated solution of Si(OH)_4_.

The super-saturated levels of Si(OH)_4_ in xylem sap of some plants have often been used as evidence for silicon transporters and the active uptake of Si(OH)_4_ from soil water (Liang et al., 2005, 2006). If the entry of Si(OH)_4_ into a plant depended only upon the establishment of an osmotic gradient, as is the case when oocytes, for example, are used to measure Si(OH)_4_ uptake in model systems, then super-saturation of Si(OH)_4_ on one side of a biological membrane would support, if not confirm, the active uptake of Si(OH)_4_. However, since the movement of Si(OH)_4_ into root and subsequently xylem follows water, and is not dependent upon an osmotic gradient, the concentration of Si(OH)_4_ in xylem will actually reflect differences between rates of movement of solute [Si(OH)_4_] and solvent (water) into, within and out of xylem tissue. For example, consider an under-saturated solution of Si(OH)_4_ (e.g., 0.5 mM) being pumped across a membrane which allowed the passage of water at rate X and the passage of the much larger Si(OH)_4_ molecule at a rate of X/10. This would result in a concentration of Si(OH)_4_ of *ca.* 5 mM in the environment immediately preceding the Si(OH)_4_-selective membrane. The combination of hydraulic force and a membrane which resists the unrestricted passage of Si(OH)_4_ will result in a soil water that was under-saturated with respect to the solubility of Si(OH)_4_ becoming super-saturated within a plant compartment, for example, xylem tissue. While Si(OH)_4_ is a small molecule it is substantially larger than water and the resistance offered to its movement *in planta* will subsequently be higher than it is to the flow of water. The ‘resistors’ in this circuitry will include the wide variety of plant aquaporins which together with other pores and channels will contribute significantly to the concentration of Si(OH)_4_ within membrane-limited compartments of, for example, xylem tissue. For any given plant species, and hence any given combination of resistors including plant aquaporins, or Si(OH)_4_ resistors, the concentration of Si(OH)_4_ in xylem will be constant for any specific soil water Si(OH)_4_ level.

The first step toward a plant being classified as a silica accumulator must be the establishment of a super-saturated concentration of Si(OH)_4_ in xylem. The extent to which this is achieved will depend upon the resistance-free entry of Si(OH)4 into xylem in combination with Si(OH)4 resistors in other areas of the plant from the root to the shoot. In plants which are not known as silica accumulators there may be Si(OH)_4_ resistors preventing its movement into xylem (silica may still be deposited in the root) or the Si(OH)_4_ resistors throughout the plant do not offer sufficient resistance to the movement of Si(OH)_4_ (relative to water) to support its concentration to a super-saturated level. Such plants may show significant silica deposition when grown in soil solutions containing high levels of Si(OH)_4_ and almost no evidence of silicification in media which are deficient in Si(OH)_4_. So, if a plant has the potential to produce a super-saturated solution of Si(OH)_4_ in xylem tissue across a wide concentration of soil water Si(OH)_4_ then it is likely to be a known silica accumulator. Some plants may only silicify at high concentrations of soil water Si(OH)_4_ and other plants may not deposit silica at all or only deposit silica in the roots. It is probably the case that most plants have the potential for biological silicification and it is the second step, the templating of the silica deposition process which discriminates between those which are highly silicified, such as horsetail, and the rest.

## Templating Silicification

Once a super-saturated concentration of Si(OH)_4_ is maintained in xylem a steady supply of hydraulically and osmotically driven Si(OH)_4_ will be available to the rest of the plant tissues. Hydrostatic and osmotic forces drive the radial and axial movement of water from xylem and in following water out of xylem vessels super-saturated Si(OH)_4_ will encounter various new compartments some of which will support time-dependent formation of dimers, trimers, oligomers, and polymers of Si(OH)_4_ and eventually the precipitation of silica. These ‘compartments’ are created by various resistors which influence the relative rates of movement of water and Si(OH)_4_ (water faster than Si(OH)_4_) and will include aquaporin-like channels, plasmodesmata and various precursors and constituents of plant tissues, such as those which constitute plant cell walls. The precise nature and abundance of such compartments will be species-specific and the degree to which they may become silicified in any one species will depend upon the soil water content of Si(OH)_4_ and the extent to which it becomes super-saturated in xylem (and perhaps analogous water conducting tissues).

**FIGURE 1:**
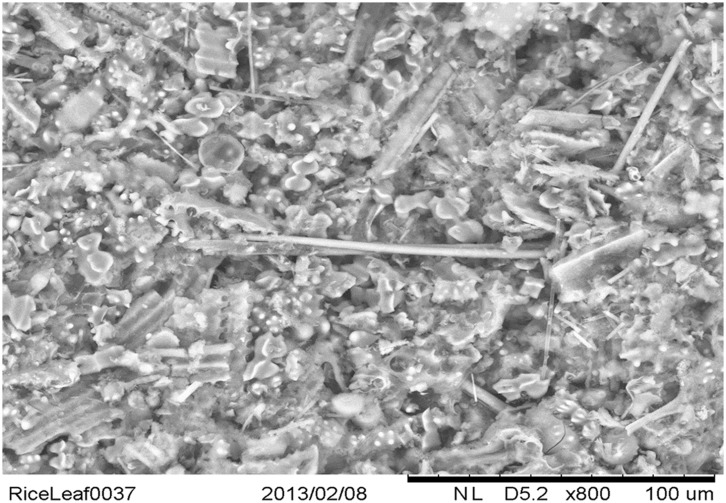
**Scanning electron microscopy image of silica collected following acid and microwave digestion of rice leaf blade and demonstrating myriad silicified structures**. The silica tube at the center of the image is approximately 100 μm in length and 4 μm in diameter.

What may not be generally appreciated is that in plants that are considered as silicon accumulators, for example horsetail and rice, silicification is extensive (Cooke and Leishman, 2011) and the degree to which tissues are silicified cannot always be appreciated using some methods of biological imaging (**Figure 1**). Biogenic silica is extraordinarily stable in acid. When silica-rich plant tissues are digested using a microwave oven at 180°C and 1800W in a 1:1 combination of 15.8M HNO_3_ and 18.4M H_2_SO_4_ and the resulting clear digests are diluted with ultra pure water and filtered through 0.10 μm membranes the only residue collected by the filters is biogenic silica. When the silica is viewed using the fluor PDMPO and fluorescence microscopy the images obtained are spectacular and in particular they emphasize the myriad structures which are silicified (Law and Exley, 2011). There are structures which appear more heavily silicified than others and their propensities for silicification are probably determined by the respective densities of the molecular structures acting as templates of the precipitation process. We have identified the hemicellulose callose as one such molecular template for biological silicification (Law and Exley, 2011) and others will probably include precursors to and components of plant cell walls (Fleck et al., 2011; Fernández Honaine and Osterrieth, 2012; Yamanaka et al., 2012; Fernández Honaine et al., 2013; Leroux et al., 2013; Zhang et al., 2013).

**FIGURE 2:**
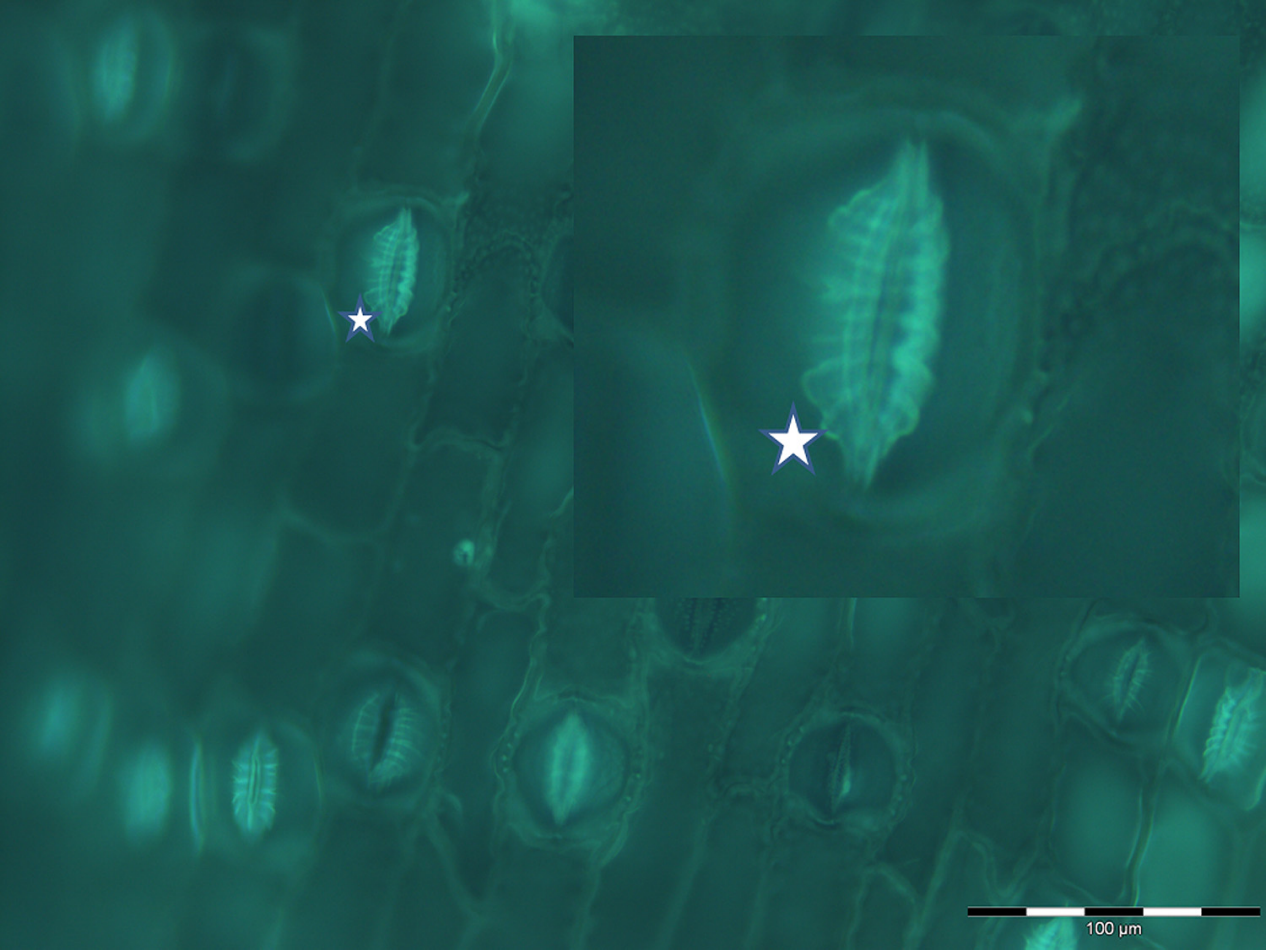
**Fluorescent imaging of silica collected following acid and microwave digestion of fern leaf (*Asplenium nidus* L.) and showing multiple stomata in silicified leaf tissue undergoing differentiation**. In particular this image, magnified in the insert, demonstrates how closely silica deposition mimics the deposition of radial fibrillar callose arrays (for example, indicated by star) in stomata in fern (Apostolakos et al., 2009).

The mechanism by which callose templates the precipitation of biogenic silica is likely to be entirely passive. Callose is as an amorphous gel-like polymer of glucose units linked by glycosidic bonds and the disorder and flexibility in its structure *in vivo* lends itself to its many functions in plants, including algae, as well as in yeasts, fungi and lichens (Piršelová and Matušíková, 2013). In plants its intracellular transport is in vesicles and it is continually synthesized and degraded by callose synthases and β-1,3-glucanases, respectively. The adaptability of callose, relative for example, to the more rigid structure of cellulose, makes it ideal as a building material for example in the differentiation of stomata or the development of plasmodesmata. The structure of callose, essentially a loose gel which is rich in hydroxyl functionalities, also makes it an ideal candidate material to provide a constrained environment to template the precipitation of Si(OH)_4_ as biogenic silica. By way of an example, the differentiation of stomata is a complex process in which callose is involved in almost every step. Apostolakos et al. (2009, 2010) have detailed these stages in fern (*Asplenium nidus* L.), a known silica accumulator (Leroux et al., 2013) and we have shown that silica deposition exactly mimics callose deposition in horsetail (Law and Exley, 2011) and in fern (**Figure 2**). These observations not only support a specific role for callose in silica deposition they demonstrate that the deposition of silica in plants is not simply a one-way process but must involve the modeling, dissolution, and remodeling of silica structures.

## Natural Selection and Plant Silicification

Silicification has conferred a range of advantages on silica accumulators and specifically structural support (Hodson et al., 2005), defense against pathogens (Ma, 2004), defense against herbivory (McNaughton et al., 1985), alleviation of micronutrient deficiency (Hernandez-Apaolaza, 2014) and amelioration of metal toxicity (Epstein, 1994). However, the propensity to support the process of silicification, the conversion of a super-saturated solution of Si(OH)_4_ to amorphous hydrated silica, may also have dictated the success of certain plants to thrive in soil solutions rich in Si(OH)_4_. To understand what is meant here it needs to be appreciated that saturated solutions of Si(OH)_4_ which are undergoing rapid auto condensation to form silica nanoparticles are known to be cytotoxic, for example causing rapid haemolysis of red blood cells (Margolis, 1961). Margolis, who described this effect, suggested that the mechanism involved the adsorption and denaturation of a globular protein and that the effect was size-specific and was only observed when silica particles exceeded 5 nm in size (Iler, 1979). Generally the auto condensation of Si(OH)_4_ is not an issue in biota, it is simply not occurring, and it is only significant in the biosilicifiers and they must achieve the formation of silica without suffering any cytotoxic effects. This suggests two prerequisites to achieving successful and toxicity-free biological silicification; (i) during early stages the size of silica nanoparticles must be maintained below 5 nm and (ii) the assembly of silica structures and frameworks involving silica particles larger than 5 nm must involve biomolecular templates which are not prone to denaturation (perhaps precluding a role for proteins?) or biomolecules which will be sacrificed as part of the silicification process. As mentioned previously, the hemicellulose, callose may be an ideal vehicle for the entrapment of Si(OH)_4_ and the subsequent control of its auto condensation and growth toward nanoparticles (<5 nm) of silica. It has the approximate structure of a sponge being able to soak up Si(OH)_4_ into myriad constrained spaces each dense with hydroxyl functionality from its constituent glucose units. While the formation of silica may be allowed within these spaces its growth will probably be significantly delayed or constrained. As was alluded to earlier the extremely detailed way in which silica deposition appears to mirror the role of callose in the differentiation of stomata, cytokinesis and the structure of plasmodesmata (Law and Exley, 2011) would suggest significant plasticity within the callose-silica system with silica both forming and dissolving to mimic the role of callose in these processes. Biological silicification is not a one-way process as it is known to be reversible to a significant extent when the source of Si(OH)_4_ to the organism is removed (Law and Exley, 2011; Yamada et al., 2014). It is a highly dynamic process and the processes which underlie callose biochemistry may also underlie biological silicification but only in those plants where a super-saturated concentration of Si(OH)_4_ is maintained in xylem and, perhaps, other conducting tissues. By way of contrast those plants which maintained a super-saturated concentration of Si(OH)_4_ in xylem tissue but did not also utilize callose (or equivalent biomolecule) have already been selected out of those environments which today support biosilicifiers.

## Step by Step Guide to Biological Silicification in Plants

The biologically available form of silicon in soil waters is Si(OH)_4_ and it follows water into the plant root.

The relative rates of movement of solute [Si(OH)_4_] and solvent (water) into xylem and other conducting tissues under hydrostatic pressure are governed by water channels, such as aquaporins, and not transporters. Where these channels present significant resistance to the movement of Si(OH)_4_, relative to water, the solute is progressively concentrated with the result that some plants maintain a super-saturated concentration of Si(OH)_4_ in these tissues, the degree of super-saturation being governed by the concentration of Si(OH)_4_ in soil water.

Super-saturated Si(OH)_4_ in the vascular system acts as a source of Si(OH)_4_ to all other tissues. Some of this Si(OH)_4_ leaves the plant through guttation. Transcellular movement of Si(OH)_4_ following concentration gradients will result in auto condensation of Si(OH)_4_ upon entering constrained environments, for example, such as those presented by the vesicular transport of callose. Silicification piggy-backing the metabolism and deposition of callose presents sophisticated cellular machinery for the controlled and specific deposition of biogenic silica. This is evident in the highly specialized silicification seen in horsetail and other biosilicifiers. However, silicification is significantly more widespread throughout plant tissues than is generally appreciated and other constrained environments, usually created by biomolecules involved in structures associated with cell walls, will also promote biological silicification to differing extents and degrees of sophistication depending upon the substrate and the delivery of Si(OH)_4_.

Silicification is a passive process in that it occurs simply as a consequence of biochemistry and cellular machinery which evolved to fulfill entirely different requirements, such as the movement of water and the differentiation of cell walls. We know that this is true as while silicification does confer advantage on some organisms it is not essential for any organism. For example, horsetail grows perfectly well in the complete absence of silica deposition in its tissues though such silica-free plants are more prone to fungal infection (Fauteux et al., 2005; Law and Exley, 2011). There is no known silicon biochemistry (Exley, 1998) and there is a simple reason for this in that the biologically available form of silicon, Si(OH)_4_, has no organic chemistry and an extremely limited inorganic chemistry. These simple facts explain the non-selection of silicon in the biochemistry of life (Exley, 2009a).

## Conflict of Interest Statement

The author declares that the research was conducted in the absence of any commercial or financial relationships that could be construed as a potential conflict of interest.
